# Development of a Novel Quantitative PCR Assay for Detecting *Schistosoma japonicum* in Field-Captured Rodents

**DOI:** 10.3390/pathogens15080825

**Published:** 2026-08-06

**Authors:** Chao Lv, Jiqin Jia, Xiaojuan Xu, Shizhen Li, Qi Sun, Jing Xu, Lijun Lin, Qunxiu Liu, Yang Hong

**Affiliations:** 1 National Key Laboratory of Intelligent Tracking and Forecasting for Infectious Diseases; National Institute of Parasitic Diseases, Chinese Center for Disease Control and Prevention (Chinese Center for Tropical Diseases Research); National Health Commission Key Laboratory of Parasite and Vector Biology; WHO Collaborating Centre for Tropical Diseases; National Center for International Research on Tropical Diseases, Ministry of Science and Technology, Shanghai 200025, China; lvchao@nipd.chinacdc.cn (C.L.); lisz1@nipd.chinacdc.cn (S.L.);; 2 College of Life Sciences, Shanghai Normal University, Shanghai 200025, China; jjq15532300528@163.com; 3 Anhui Provincial Center for Disease Control and Prevention, Hefei 230601, China; 4 Center for Disease Control and Prevention of Wei Hai, Weihai 264200, China; sunqiqi@yeah.net; 5 School of Basic Medicine and Forensic Medicine, Hangzhou Medical College, Hangzhou 311300, China; 6 School of Health and Social Care, Shanghai Urban Construction Vocational College, Shanghai 201415, China

**Keywords:** *Schistosoma japonicum*, Quantitative PCR, field-captured rodents, surveillance

## Abstract

In the context of the current low endemicity of schistosomiasis in China, *Schistosoma japonicum* (*S. japonicum*) infections increasingly have been reported in field-captured rodents, highlighting the need for rapid and scalable tools to support surveillance during the elimination stage. This study developed and evaluated a quantitative polymerase chain reaction (qPCR) assay targeting a highly repetitive *S. japonicum* sequence for detecting infection in liver tissues. Analytical performance was assessed using adult-worm genomic DNA, DNA from non-target pathogens, egg-spiked mouse liver samples, experimentally infected BALB/c mice, and liver samples from field-captured rodents collected in Dongzhi and Duchang counties. The optimized assay generated a single melting peak at 79 ± 0.5 °C, used Ct < 33 as the positivity cutoff, and showed no apparent cross-reactivity with the non-target pathogens tested. Using serially diluted adult-worm genomic DNA, the assay consistently detected *S. japonicum* DNA at concentrations as low as 1 pg/μL. In the egg-spiked liver model, as few as two eggs were detected in 200 mg of liver tissue. In the experimentally infected model, all infected mice tested positive, whereas all non-infected controls tested negative, indicating complete agreement between the qPCR results and the experimentally defined infection status. In the field evaluation, qPCR yielded a higher positivity rate than parasitological examination in Dongzhi County (66.81% vs. 51.72%) and identified six positive samples in Duchang County, where all samples were negative by parasitological examination. These findings indicate that the qPCR assay can facilitate sensitive and high-throughput detection of *S. japonicum* infection in field-captured rodents and may provide a useful complementary tool for surveillance during schistosomiasis elimination.

## 1. Introduction

Schistosomiasis, also known as bilharzia, is an acute and chronic helminthic disease caused by infection with blood flukes of the genus *Schistosoma*. As one of the most important parasitic diseases worldwide, schistosomiasis remains endemic across 78 countries and territories, placing an estimated 800 million people at risk of infection and imposing a substantial global disease burden [1,2]. According to Global Burden of Disease (GBD) 2021, the burden of schistosomiasis is disproportionately concentrated in low Socio-demographic Index regions with limited health-system capacity and insufficient public health resources, particularly Western sub-Saharan Africa, Eastern sub-Saharan Africa, and East Asia [3]. This persistent and inequitable burden may further impede progress toward the United Nations Sustainable Development Goals, particularly those related to health, poverty reduction, education, clean water and sanitation, and economic productivity [4]. Among the six *Schistosoma* species known to infect humans worldwide, *Schistosoma japonicum* (*S. japonicum*) causes schistosomiasis japonica, which is mainly endemic in China, the Philippines, and Indonesia [5]. After decades of sustained control efforts, the prevalence of schistosomiasis in China has declined markedly, and newly detected infections in humans and livestock have become increasingly rare [6,7,8]. Nevertheless, given the natural-focal and zoonotic nature of schistosomiasis japonica, sustained surveillance remains necessary even after interruption of transmission or achievement of elimination targets.

Compared with other human-infecting *Schistosoma* species, *S. japonicum* has a broader definitive-host spectrum, with documented infections in more than 40 mammalian hosts, including humans, livestock, and field-captured rodents [9,10,11,12]. In most endemic areas of China, humans and livestock have traditionally been regarded as the principal sources of *S. japonicum* transmission, and control efforts have therefore primarily targeted these two reservoir host groups [7,13,14]. Recently, enhanced surveillance has revealed concerning evidence of *S. japonicum* infection in field-captured rodents in certain endemic areas, with prevalence rates exceeding 20% [12,15,16]. These findings are unprecedented after nearly a decade of intensive schistosomiasis control efforts in China and suggest that field-captured rodents may represent an important source of transmission at the present stage. Importantly, the relatively high infection rate in field-captured rodents may pose a serious challenge that could undermine progress toward schistosomiasis elimination in China [17,18]. Accordingly, this potential threat underscores the urgent need for comprehensive investigations, including field epidemiological surveys, laboratory detection, and genetic analyses. In particular, the accurate detection and characterization of infected rodent populations are essential for evaluating their epidemiological significance and informing targeted surveillance and control strategies.

Previous studies have described key epidemiological features of *schistosome* infection in field-captured rodents, including field-survey designs and infection detection methods [15,16]. However, the detection has relied predominantly on parasitological techniques, with molecular methods used only to a limited extent. Notably, Liver tissue has been identified as an important specimen for detecting *S. japonicum* infection in field-captured rodents because tissue-retained eggs can provide direct evidence of infection [19]. This sampling approach also provides a basis for developing diagnostic methods with improved sensitivity and higher throughput. Although parasitological methods remain the gold standard for definitive diagnosis of schistosomiasis, their labor-intensive workflow and reliance on specialist expertise make them impractical for large-scale surveys, particularly in the context of low-intensity infection [20,21]. In contrast, molecular assays are easier to standardize, require less specialized training, and offer higher sensitivity for early infections, with the added potential for rapid pattern. To date, multiple molecular detection methods targeting diverse genetic markers have been developed for *S. japonicum*, including 18S rRNA, 28S rRNA, *cox1*, NADH I, and SjR2. These approaches, such as conventional PCR, quantitative PCR (qPCR), loop-mediated isothermal amplification (LAMP), and recombinase polymerase amplification (RPA), have shown utility in laboratory diagnosis, and some have been further applied in field-based surveys and surveillance [22].

Nevertheless, most published molecular assays have been developed and evaluated primarily using samples from humans, livestock, snails, feces, or environmental sources, whereas their application to wild rodent liver specimens remains limited. Moreover, many existing targets were selected from conserved or empirically identified genomic regions and may not be optimal for detecting low-burden infections in liver tissues with abundant host-derived DNA. Therefore, developing an additional assay based on a newly identified, high-copy, and species-specific molecular marker may provide a useful technical reserve and expand the diagnostic options available for wildlife surveillance. To address the urgent need for a reliable, high-throughput detection molecular method of *S. japonicum* infection in field-captured rodents, a qPCR assay targeting a novel molecular marker was developed and evaluated for its applicability to ongoing field investigations using liver specimens from previous field investigations.

## 2. Materials and Methods

### 2.1. Genomic DNA Extraction from Adult S. japonicum Worms

Adult *S. japonicum* worms were collected from infected BALB/c mice which were experimentally infected with cercariae through percutaneous exposure of the abdominal skin, which mimics the natural route of *schistosome* infection, and stored in RNAlater (Sigma-Aldrich, St. Louise, USA) at −20 °C until use. Genomic DNA of adult worms was extracted using a commercial kit (TIANGEN, Beijing, China) following the manufacturer’s instructions. DNA concentration was measured with a NanoDrop 2000 Spectrophotometer (Thermo Fisher Scientific, Waltham, MA, USA) and diluted to 20 ng/μL with nuclease-free water.

### 2.2. Establishment of a qPCR Assay for Detecting

A highly repetitive target sequence specific to *S. japonicum* was selected through bioinformatics analysis of the *S. japonicum* genome. Briefly, genome assemblies of representative *Schistosoma* species were mined to identify species-specific, high-copy-number transposable element targets. Candidate sequences were filtered by comparative genomic analysis, experimentally validated by qPCR for species specificity, and then incorporated into a one-tube RPA–CRISPR/Cas12a detection system. The assay was evaluated using recombinant plasmids, genomic DNA from multiple parasite species, and diverse biological samples, including serum, feces, snail, and fish tissues, to assess its sensitivity, specificity, and applicability across different host sources and parasite life-cycle stages.

Then, PCR primer pairs were designed with consideration of amplicon length, primer melting temperature, GC content, and the absence of predicted primer-dimers or nonspecific amplification using Beacon Designer version 2.1 (PREMIER Biosoft International, Palo Alto, CA, USA). The design criteria included a primer length of 18–25 nucleotides, a target melting temperature of approximately 60 °C, a maximum melting-temperature difference of 2 °C between the forward and reverse primers, a GC content of 40–60%, and an expected amplicon length of 70–200 bp. Candidate primers with predicted hairpin structures, self-dimers, cross-dimers, long homopolymeric stretches, or significant off-target homology were excluded. Primer pairs receiving the highest software ratings were preferentially selected and subsequently verified using BLAST 2.16.0 analysis. The final primers were selected and listed below:

Forward: 5’-CAAATACACAGTCTTTTCCCGTTTC-3’,

Reverse: 5’-AGGTCCAGAGTTTACAGTGCTAAG-3’.

A 20 μL qPCR reaction was set up using *S. japonicum* genomic DNA as the template. Each reaction mixture contained 10 μL of 2× ChamQ SYBR qPCR Master Mix (Vazyme, Nanjing, China), 0.4 μL each of forward and reverse primers (final 0.2 μM), 1 μL of DNA template (20 ng/μL), and 8.2 μL of nuclease-free water. Amplification was performed on a CFX96 Real-Time PCR system (Bio-Rad, Hercules, CA, USA) with fluorescence acquisition in the FAM channel. The optimal annealing temperature was determined by a gradient from 57 to 61 °C, and 58 °C was selected as the final temperature. The optimized thermal cycling program was: 95 °C for 5 min; 40 cycles of denaturation at 95 °C for 10 s, annealing at 58 °C for 30 s, with fluorescence signal captured at 58 °C for each cycle. Melting-curve analysis was performed from 65 °C to 95 °C, with a temperature increment of 0.5 °C and a 5 s hold at each step. Fluorescence signals were automatically acquired throughout the melting-curve analysis. Cross-reactivity testing was assessed using genomic DNA from diverse species of pathogens, including *Angiostrongylus cantonensis*, *Clonorchis sinensis*, *Echinococcus granulosus*, *Escherichia coli*, *Babesia* spp., and *Leishmania* spp. The Cycle threshold (Ct) cutoff value for distinguishing positive from negative results was determined based on the amplification curves and melting-curve profiles of *S. japonicum* genomic DNA and other pathogen controls.

### 2.3. Evaluation of the Detection Limit of the qPCR Assay Using Serially Diluted Genomic DNA

To evaluate the analytical sensitivity and reproducibility of the established qPCR assay, genomic DNA extracted from adult *S. japonicum* worms was used to prepare a serial dilution panel. The genomic DNA was first adjusted to 100 ng/μL with nuclease-free water and then subjected to ten-fold serial dilution to generate 10 concentration gradients. For each concentration, three parallel dilution replicates were prepared and tested using the established qPCR assay. To assess inter-plate reproducibility, the entire dilution panel was tested again on a separate qPCR plate under the same reaction conditions. No-template controls were included in each run. The detection limit was defined as the lowest DNA concentration that consistently produced a positive amplification signal with a Ct value below the predefined cutoff and a specific melting peak.

### 2.4. Evaluation of the qPCR Assay Using an Egg-Spiked Liver Model

Purified *S. japonicum* eggs stored in our laboratory were used for the egg-spiked liver model. These eggs had been prepared from the livers of multiple experimentally infected mice at 42 or 48 days post-infection. The infected livers were homogenized and then sequentially passed through copper sieves with different mesh sizes. The material retained on the 260-mesh copper sieve was collected and subjected to repeated centrifugation to remove the upper layer of liver debris. The pellet was then resuspended in 1.2% saline and centrifuged repeatedly until purified eggs were obtained.

Purified *S. japonicum* eggs were added to mouse liver tissues to establish an egg-spiked liver model with six gradients, corresponding to 0, 2, 5, 10, 20, and 40 eggs per 200 mg liver tissue sample. After genomic DNA extraction, the samples were tested using the qPCR assay described above to evaluate the performance of the assay in detecting parasite eggs in liver tissue.

### 2.5. Evaluation of the qPCR in Infected BALB/c Mouse Model

BALB/c mice were individually infected with 5, 10, 20, or 40 cercariae. Liver samples were collected before infection and at 28, 35, and 42 days post-infection. Infection status was determined by examining pathological changes on the liver surface and by recovering parasites from the portal and mesenteric veins. Each infection group comprised eight mice, with two mice euthanized for liver collection at each time point. A 200 mg liver tissue sample was homogenized, and then genomic DNA was extracted. DNA concentrations were quantified, and the qPCR was performed as described above, with each sample analyzed in triplicate.

To preliminarily evaluate the sensitivity and specificity of the established qPCR assay under controlled experimental conditions, an additional set of experimentally infected and negative control mice was included. Ten mice infected with 10 cercariae and euthanized at 42 days post-infection, together with 10 negative control mice, were selected for analysis. For each mouse, 200 mg of liver tissue was randomly collected, homogenized, and subjected to genomic DNA extraction. The extracted DNA was then tested using the established qPCR assay to preliminarily evaluate the sensitivity and specificity of the method.

### 2.6. Collection of Field-Captured Rodents and Parasitological Detection

A total of 232 liver samples from field-captured rodents were collected in Dongzhi County, Anhui Province, and 74 liver samples were collected in Duchang County, Jiangxi Province. Both counties are historically endemic areas for schistosomiasis in China and have continued value for field surveillance. These two counties were selected because previous field investigations had detected *S. japonicum* infection in field-captured rodents in these areas, ensuring that the available samples included positive specimens suitable for evaluating the detection performance of the qPCR assay. In addition, previous surveillance suggested that the two counties represented different field settings, with a relatively high positivity rate in field-captured rodents in Dongzhi County and a lower positivity rate in Duchang County, allowing a preliminary assessment of assay performance under different infection-level conditions.

Among the 232 field-captured rodents captured in Dongzhi County, 117 were captured in June across two villages, and the remaining rodents were captured in September in the same villages. In Duchang County, 36 field-captured rodents were captured in June, and 38 were captured in September in two selected villages. Several rodent species were captured during the field investigations; however, most samples in both counties were obtained from *Rattus norvegicus*.

### 2.7. Detection of Wild Rodent Liver Samples by qPCR

A total of 200 mg of liver tissue from each rodent was homogenized, and genomic DNA was extracted using the same commercial tissue DNA extraction kit (TIANGEN, Beijing, China). The DNA concentration was also measured by a NanoDrop 2000 Spectrophotometer. Using the established qPCR assay, all wild rodent samples were tested, with each sample analyzed in technical triplicate. Positive and negative controls were included in each detection. The qPCR positivity rate was calculated and compared with parasitological results to assess concordance. Subsequently, using parasitology results as the reference, the accuracy of the qPCR assay was then evaluated.

### 2.8. Statistical Analysis

Chi-square tests were used to compare rodent positivity rates across regions and sampling months, and consistency between parasitological and qPCR results was evaluated. Because the primary objective of this study was to assess the detection performance and field applicability of the qPCR assay rather than to compare infection rates among different rodent species, species-stratified epidemiological analyses were not performed. A paired Student’s *t*-test was used to compare Ct values obtained from the same DNA concentration across independent qPCR plates, thereby assessing inter-plate Ct-value variation. A significance level of α = 0.05 was used for the aforementioned analyses. All statistical analyses were conducted in R v4.3.3.

## 3. Results

### 3.1. Establishment of the qPCR Assay

The qPCR assay yielded typical amplification curves in the FAM channel. A gradient annealing temperature test (57–61 °C) identified 58 °C as the optimal temperature. Under this condition, melt-curve analysis showed a single peak (Tm = 79 ± 0.5 °C) with no primer dimers and nonspecific products. With the positivity criterion set as Ct < 33 together with the specific melt peak, no amplification was observed for genomic DNA (20 ng/μL) from non-target species, including *Angiostrongylus cantonensis*, *Clonorchis sinensis*, *Echinococcus granulosus*, *Escherichia coli*, *Babesia* spp., and *Leishmania* spp., indicating no apparent analytical cross-reactivity with the non-target organisms tested in this study.

### 3.2. Detection Limit of the qPCR Assay Using Serially Diluted S. japonicum Genomic DNA

The qPCR assay consistently detected serially diluted *S. japonicum* genomic DNA from 100 ng/μL to 1 pg/μL on two independent qPCR plates. The mean Ct values were 13.63 ± 0.16, 20.19 ± 0.05, 22.39 ± 0.14, 25.54 ± 0.16, 29.05 ± 0.34, and 32.29 ± 0.63 at DNA concentrations of 100 ng/μL, 10 ng/μL, 1 ng/μL, 100 pg/μL, 10 pg/μL, and 1 pg/μL, respectively. At 1 pg/μL, all technical replicates remained positive, whereas at 100 fg/μL and lower concentrations, amplification was inconsistent or the Ct values exceeded the predefined positivity cutoff of 33. Therefore, the analytical detection limit of the assay was determined to be 1 pg/μL. Paired Student’s *t*-test showed no significant difference in mean Ct values between the two plates within the reliable detection range (t = 2.074, *p* = 0.093), indicating good inter-plate reproducibility.

### 3.3. Detection Performance of the qPCR Assay in an Egg-Spiked Liver Model

In the egg-spiked liver model, the blank control containing 0 eggs per 200 mg of liver tissue showed an average Ct value of 36.37 ± 1.01, which was above the predefined positivity cutoff of Ct = 33 and was therefore considered negative. Liver tissue samples spiked with 2, 5, 10, 20, and 40 eggs were all detected as qPCR positive, with average Ct values of 32.71 ± 0.33, 32.67 ± 0.28, 31.68 ± 0.28, 32.64 ± 0.25, and 31.10 ± 0.09, respectively. Overall, Ct values tended to decrease as the number of spiked eggs increased, with the lowest Ct value observed in the 40-egg group. These results indicate that the qPCR assay could detect as few as 2 *S. japonicum* eggs in 200 mg of liver tissue, suggesting good detection performance in liver samples with low egg burdens.

### 3.4. The Result of the qPCR on Detecting BALB/c Mouse Samples

BALB/c mice were infected with 5 cercariae as a low-intensity infection model, 10 or 20 cercariae as moderate-intensity infection models, and 40 cercariae as a high-intensity infection model. At 28, 35, and 42 days post-infection, all liver samples (n = 2 mice at each time point) tested positive by qPCR. Ct values displayed a dose-dependent inverse relationship with inoculum size, with higher parasite burdens corresponding to lower Ct values. No clear association was observed between Ct values and time post-infection (Table 1).

The detection performance of the qPCR assay was further evaluated using liver tissue samples from infected mice and negative control mice. All liver samples from the 10 non-infected mice showed Ct values above the cutoff of Ct = 33, with a range of 33.68 ± 0.81 to 34.84 ± 0.64, and were therefore classified as qPCR negative. In contrast, all liver samples from the 10 mice infected with 10 cercariae and sampled at 42 days post-infection were qPCR positive, with a range of 18.32 ± 0.98 to 26.46 ± 0.31. The qPCR results showed complete agreement with the experimentally defined infection status, with all infected mice testing positive and all non-infected mice testing negative, corresponding to an overall agreement of 100% in this experimental model (Table 2).

### 3.5. Field Performance in Dongzhi County, Anhui Province

Among 232 wild-rodent liver samples, 155 were qPCR positive, resulting in a positivity rate of 66.81% (155/232), which was significantly higher than that determined by parasitology (51.72%, 120/232, χ^2^ = 10.936, *p* < 0.001). In Village 1 (n = 131), the qPCR positivity rate was 74.05% (97/131) versus 51.15% (67/131) by the parasitological method (χ^2^ = 14.671, *p* < 0.001). In Village 2 (n = 101), the qPCR positivity rate was 57.43% (58/101) versus 52.48% (53/101) by the parasitological method (χ^2^ = 0.500, *p* = 0.480). Furthermore, qPCR detected a significant inter-village difference (χ^2^ = 7.104, *p* = 0.008), whereas no such difference was found with the parasitological method (χ^2^ = 0.040, *p* = 0.841). These results indicate that the qPCR assay offers greater discriminatory power for spatial risk assessment of schistosomiasis transmission (Table 3).

For field-captured rodents captured in June, the qPCR positivity rate was 77.78% (91/117), which was significantly higher than that determined by the parasitological method (48.72%, 57/117; χ^2^ = 21.253, *p* < 0.001). For field-captured rodents captured in September, the qPCR positivity rate was 55.65% (64/115), which showed no significant difference with that of the parasitological method (54.78%, 63/115; χ^2^ = 0.018, *p* = 0.895). When positivity rates were compared between sampling months, qPCR detected a significantly higher infection rate in June than in September (77.78%, 91/117 vs. 55.65%, 64/115; χ^2^ = 12.804, *p* < 0.001), whereas no significant temporal difference was observed using the parasitological method (48.72%, 57/117 vs. 54.78%, 63/115; χ^2^ = 0.854, *p* = 0.355). These findings suggest that qPCR may be more sensitive in detecting temporal variation in *S. japonicum* infection among field-captured rodents.

### 3.6. Field Performance in Duchang County, Jiangxi Province

Among 74 wild rodent samples, qPCR detected six positive samples, with a positivity rate of 8.11% (6/74), whereas all samples tested negative by the parasitological method. All 36 samples collected in June were negative by both methods, and the six qPCR-positive samples were identified from rodents captured in September.

### 3.7. Consistency Test (Samples from Dongzhi County)

The consistency analysis was performed using 232 samples from Dongzhi County. Compared with the parasitological method, the qPCR assay yielded an overall accuracy of 68.53% (159/232), with a Cohen’s kappa value of 0.363, which was a low level of consistency between the two tests. Accuracy was similar across different subgroups, with values of 69.47% (kappa = 0.383) and 67.32% (kappa = 0.342) in Village 1 and Village 2, respectively, and 69.23% (kappa = 0.393) and 67.83% (kappa = 0.349) for June and September samples, respectively (Table 4). In addition, one case of single Capillaria hepatica infection was identified in the liver by parasitological examination, and this sample tested negative by the qPCR assay.

## 4. Discussion

The persistence of *S. japonicum* infection in field-captured rodents, characterized by high prevalence in some regions, poses a critical impediment to schistosomiasis elimination in China [11,14,15,18]. This threat is amplified by the continued and widespread presence of the *Oncomelania hupensis* snail, which was the only intermediate host of *S. japonicum* habitats in endemic areas [23]. Consistent with the emphasis on wildlife surveillance in China’s latest national schistosomiasis control plan, there is a pressing need for diagnostic methods that are rapid, accurate, and scalable [24]. To address this gap, a qPCR assay was developed that can complete the reaction and generate interpretable results within 1.5 h. The assay demonstrates superior performance, showing higher positivity rates than parasitological examinations and low cross-reactivity with other species of pathogens. With further optimization, this approach could become highly valuable for ongoing field surveillance.

The present qPCR assay consistently detected *S. japonicum* genomic DNA at a concentration of 1 pg/μL, demonstrating its capacity to detect low concentrations of purified parasite DNA. qPCR assays targeting other markers, including NADH I [25,26,27,28,29,30,31,32], 18S rRNA [33,34,35,36], SjR2 [34,37,38], ITS2 [39] and Sjrh1.0 [34], likewise achieved detection limits in the picogram or even femtogram range. Notably, an 18S rRNA-targeting qPCR assay reported a genomic DNA detection limit as low as 0.4 fg [35]. Nevertheless, differences in target copy number, primer and probe design, amplification chemistry, and reaction conditions should be considered when comparing analytical detection limits across assays. In the egg-spiked liver model, the assay detected as few as two eggs in 200 mg of liver tissue, equivalent to 10 eggs per gram of tissue. This level of detection was broadly comparable to those reported for qPCR assays targeting other genomic regions, although some NADH I-based assays have detected as little as one egg per gram of feces [25,26,28,29]. This comparison should also be interpreted cautiously because liver tissue and feces differ substantially in egg distribution, background host DNA, PCR inhibitors, and DNA extraction efficiency. The field evaluation further supported the potential utility of the assay. In Dongzhi County, where infection was relatively common among the field-captured rodents, the qPCR positivity rate was 66.81% (155/232), significantly higher than the 51.72% (120/232) detected by parasitological examination. In Duchang County, where prevalence was low, qPCR identified six positive samples among 74 rodent liver specimens, whereas parasitological examination detected none. These findings are consistent with previous field evaluations of NADH I-targeting qPCR assays and support the potential application of the present method in field surveillance [27,28,29]. Compared with conventional parasitological techniques, qPCR is suitable for high-throughput testing and can substantially improve detection efficiency. It also requires less specialized training in parasite egg identification and morphological examination [22,40]. At present, DNA extraction from liver tissue remains the principal rate-limiting step. Further optimization of tissue homogenization and DNA extraction, together with automation of DNA extraction and qPCR setup, could improve the throughput, reproducibility, and wider application of the assay. Integrated automated systems have already been successfully implemented for the detection of specific pathogens in other sample types, supporting the feasibility of this approach [41,42].

In a murine infection model spanning low, medium, and high parasite burdens, the assay showed excellent performance: all samples collected at four weeks post-infection were positive, indicating robust detection across infection intensities. A key feature of qPCR is its ability to quantify DNA in a sample using a standard curve, generated either by spiking samples with a known amount of template or by employing serial DNA dilutions [22]. In our animal model, a preliminary observation was that higher infection levels corresponded with lower Ct values. This suggests that in field applications, Ct values from the same reaction could provide a preliminary indicator of infection intensity [32], although this phenomenon requires confirmation with a larger sample size.

Although qPCR outperformed the parasitological method in detecting *S. japonicum* infection in wild rodent livers across all field sites, the magnitude of the difference varied by geography, likely reflecting local infection intensities. In the high-prevalence setting of Dongzhi County, qPCR revealed significant inter-village variation that the parasitological method did not capture, supporting spatial heterogeneity in transmission and also demonstrating qPCR’s greater discriminative power for fine-scale risk mapping, even as the method further standardization is needed. Conversely, in the low-prevalence setting of Duchang County, surveillance aims primarily to identify any potential existing transmission risk. In this context, qPCR showed critical sensitivity, detecting six infections among 74 parasitology-negative rodents, supporting its role as a supplementary or alternative tool in low-prevalence areas.

A more pronounced divergence between qPCR and parasitological positivity rates was observed in June compared to September in Dongzhi County, with qPCR results also showing a significant difference between these time points. This temporal pattern indicates lower infection intensity in rodents early in the transmission season (June) than later (September). This capacity to detect positives at low infection intensities and at the onset of the transmission season provides additional evidence to justify earlier interventions [27,43].

Despite the assay exhibiting a high positivity rate and high-throughput capacity for detecting *S. japonicum* infection in field-captured rodents compared to parasitological methods, this study has several limitations that warrant consideration. First, the analytical evaluation of the assay was based primarily on serial dilutions of *S. japonicum* genomic DNA and experimentally constructed egg-spiked liver samples. Although these experiments provided an initial estimate of the analytical detection limit, no plasmid or synthetic standard containing a known number of target copies was used. Consequently, the absolute copy-number-based limit of detection and other analytical characteristics, including amplification efficiency, linear dynamic range, intra-assay precision, and inter-assay reproducibility, were not systematically evaluated. Second, while the qPCR process is rapid, the DNA extraction remains labor-intensive and time-consuming, hindering field applicability of the assay. Third, although the method showed higher detection rates, its consistency with parasitological methods was suboptimal, with false negatives observed even in samples with high fecal egg counts. The reasons were potentially attributable to both limited liver sampling representativeness and suboptimal reaction conditions. It should also be noted that the liver tissues examined microscopically were not the same portions used for DNA extraction. The qPCR assay was performed using randomly sampled liver tissue, and the 200 mg tissue sample was not generated by pooling tissue from all liver lobes. Given the potential heterogeneous distribution of eggs or parasite DNA within the liver, this sampling strategy may partly explain the discrepancies observed between parasitological examination and qPCR detection. Finally, the lack of comparative analysis with other molecular assays prevents evaluation of its relative advantages in molecular diagnostics. Future efforts should prioritize optimizing pre-analytical procedures, refining reaction conditions, transitioning to probe-based detection systems, and conducting comprehensive comparative validations. In addition, the lack of detailed rodent species information and ecological exposure data may limit the interpretation of the field epidemiological significance of our findings. Therefore, while the present study supports the applicability of the qPCR assay for detecting *S. japonicum* infection in field-captured rodents, the role of different rodent species and their potential contribution to local transmission require further investigation.

Current surveillance of *S. japonicum* infection in field-captured rodents still relies on parasitological techniques, which require skilled personnel and are not amenable to high-throughput screening. Meanwhile, molecular detection methods remain notably underutilized in this context. Building on prior bioinformatic analysis and laboratory evaluation, this study assessed a novel qPCR assay and the qPCR assay demonstrates higher detection rates and finer spatial resolution at small geographical scales. With further optimization of DNA extraction and reaction conditions to enhance accuracy, this qPCR assay could serve as a promising candidate tool for identifying *S. japonicum* infection in wild rodent populations to support surveillance needs during the elimination phase of schistosomiasis in China.

## 5. Conclusions

In this study, we developed and evaluated a qPCR assay for detecting *S. japonicum* infection in liver tissue samples, with a particular focus on its application in wild rodent surveillance. The assay showed good analytical specificity, was able to detect low concentrations of genomic DNA and low egg burdens in liver tissue, and demonstrated reliable detection performance in experimentally infected mice. In field-collected wild rodent samples, qPCR yielded higher positivity rates than conventional parasitological examination and showed better ability to identify spatial and temporal differences in infection risk. These findings suggest that the qPCR assay may serve as a useful supplementary tool for detecting *S. japonicum* infection in wild rodent populations, particularly in low-endemicity or elimination-stage settings where sensitive and scalable surveillance methods are needed. Further optimization of liver tissue sampling, DNA extraction, reaction conditions, and comparative validation with other markers and molecular assays will help define its relative performance and support the establishment of a standardized molecular diagnostic framework for *S. japonicum* surveillance in field-captured rodents.

## Figures and Tables

**Table 1:** Detection results of BALB/c mouse liver samples by qPCR.

Time After Infection	Number of Cercariae	Number of Detected Mice	Number of Positive Mice	Average Ct Value (Average ± SD)
4 weeks	5	2	2	25.59 ± 1.07
4 weeks	10	2	2	29.75 ± 1.36
4 weeks	20	2	2	26.18 ± 1.09
4 weeks	40	2	2	19.51 ± 0.79
5 weeks	5	2	2	22.39 ± 1.04
5 weeks	10	2	2	18.30 ± 0.56
5 weeks	20	2	2	19.54 ± 1.25
5 weeks	40	2	2	17.98 ± 1.16
6 weeks	5	2	2	25.82 ± 1.47
6 weeks	10	2	2	24.48 ± 1.19
6 weeks	20	2	2	19.99 ± 0.97
6 weeks	40	2	2	20.52 ± 0.71

**Table 2:** The sensitivity and specificity of the qPCR assay using BALB/c mouse model.

ID	Infection Status of Mice	Average Ct Value (Average ± SD)	Results of qPCR
N1	Non-infected	34.84 ± 0.64	Negative
N2	Non-infected	34.21 ± 0.33	Negative
N3	Non-infected	33.75 ± 0.69	Negative
N4	Non-infected	34.25 ± 1.01	Negative
N5	Non-infected	34.20 ± 1.25	Negative
N6	Non-infected	34.48 ± 0.46	Negative
N7	Non-infected	34.26 ± 0.58	Negative
N8	Non-infected	34.05 ± 1.35	Negative
N9	Non-infected	34.70 ± 0.30	Negative
N10	Non-infected	33.68 ± 0.81	Negative
P1	Infected	26.46 ± 0.31	Positive
P2	Infected	20.25 ± 0.76	Positive
P3	Infected	24.94 ± 0.21	Positive
P4	Infected	19.45 ± 0.46	Positive
P5	Infected	19.67 ± 1.01	Positive
P6	Infected	18.32 ± 0.98	Positive
P7	Infected	25.44 ± 0.65	Positive
P8	Infected	25.36 ± 0.26	Positive
P9	Infected	20.01 ± 0.44	Positive
P10	Infected	21.33 ± 1.21	Positive

**Table 3:** Detection results of Field-Captured Rodents Liver Samples by qPCR and Parasitological Methods.

Province	County	Village	June			September		
			Number of Field-Captured Rodents Captured	Number of qPCR-Positive Samples	Number of Parasitologically Positive Samples	Number of Field-Captured Rodents Captured	Number of qPCR-Positive Samples	Number of Parasitologically Positive Samples
Anhui	Dongzhi	Village 1	64	50	30	67	47	37
		Village 2	53	41	27	48	17	26
Total			117	91	57	115	64	63
Jiangxi	Duchang	Village 3	15	0	0	11	1	0
		Village 4	21	0	0	27	5	0
Total			36	0	0	38	6	0

**Table 4:** Table for Consistency Analysis Between qPCR and Parasitological Detection Methods.

Methods		Parasitological Methods		Village 1		Village 2		June		September	
				Parasitological Methods		Parasitological Methods		Parasitological Methods		Parasitological Methods	
		Negative	Positive	Negative	Positive	Negative	Positive	Negative	Positive	Negative	Positive
qPCR	Positive	54	101	35	62	19	39	35	56	19	45
	Negative	58	19	29	5	29	14	25	1	33	18
Total		112	120	64	67	48	53	60	57	52	63

## Data Availability

All data generated or analyzed during this study are included in this article.

## Abbreviations

The following abbreviations are used in this manuscript:

qPCR	Quantitative PCR
*S. japonicum*	*Schistosoma japonicum*
LAMP	loop-mediated isothermal amplification
RPA	recombinase polymerase amplification
